# A rare twist: Intussusception triggered by a jejunostomy tube

**DOI:** 10.1016/j.radcr.2025.11.050

**Published:** 2025-12-13

**Authors:** Chakir Mahfoud, Hatim Essaber, Anwar Kalali, Hamza Charif, Fatima Zahra Laamrani, Youssef Omor, Rachida Latib, Sanae Amalik

**Affiliations:** a Department of Radiology, National Institute of Oncology, Mohammed V University, Rabat, Morocco; b Department of medical Oncology, National Institute of Oncology, Mohammed V University, Rabat, Morocco

**Keywords:** Intussusception, Jejunostomy tube, Target sign, Small-bowel obstruction

## Abstract

Jejunostomy-related intussusception is an exceptionally rare postoperative complication, accounting for less than 1% of all small-bowel obstructions. It may result from mechanical irritation or altered motility around the feeding tube. We describe the case of a 63-year-old man with unresectable adenocarcinoma of the gastric cardia who underwent exploratory laparotomy with placement of a feeding jejunostomy, followed by palliative chemotherapy. A few days after surgery, he developed progressive abdominal distension and clinical signs of bowel obstruction. Abdominal CT revealed small-bowel dilatation and a characteristic “target sign’’ at the ileocecal valve, consistent with intussusception induced by the jejunostomy tube. Surgical exploration confirmed the diagnosis, and bowel resection was performed. This case underscores the importance of considering intussusception as a potential cause of postoperative bowel obstruction in patients with feeding jejunostomy and highlights the crucial role of early imaging and timely surgical intervention in preventing ischemic complications.

## Introduction

Intussusception, first described by Barbette in 1674 and later detailed by John Hunter in 1789, is an uncommon cause of bowel obstruction in adults, representing approximately 1%-5% of cases. Unlike pediatric intussusception—which is usually idiopathic and often resolves with non-operative reduction—adult intussusception is typically secondary to an identifiable pathological lead point, such as neoplasms, Meckel’s diverticulum, or inflammatory strictures. Because of the high association with malignancy, reported in up to 65% of cases, preoperative radiologic reduction is generally not recommended [1]. As a result, definitive surgical management, often involving bowel resection, remains the standard of care for most adult patients.

## Case presentation

We report the case of a 63-year-old man with no significant prior medical history who was evaluated for an infiltrative, ulcerative–proliferative adenocarcinoma of the gastric cardia. Staging investigations, including an abdominal computed tomography (CT) scan, demonstrated a malignant cardial mass with suspected invasion of the left diaphragmatic crus. Exploratory laparotomy confirmed a locally advanced, fixed tumor that was deemed unresectable due to superior plane invasion. A feeding jejunostomy was therefore placed, and palliative chemotherapy was initiated.

Several days after surgery, the patient presented to the emergency department with a 5-day history of complete cessation of flatus and bowel movements, progressive abdominal distension, and overall clinical decline. On examination, he was afebrile, with stable vital signs, and exhibited a mildly distended and tender abdomen without peritoneal signs. Laboratory tests revealed leukocytosis (12,800/µL; normal 4000–10,000/µL) and mildly elevated C-reactive protein (18 mg/L; normal <5 mg/L).

**Fig. 1:**
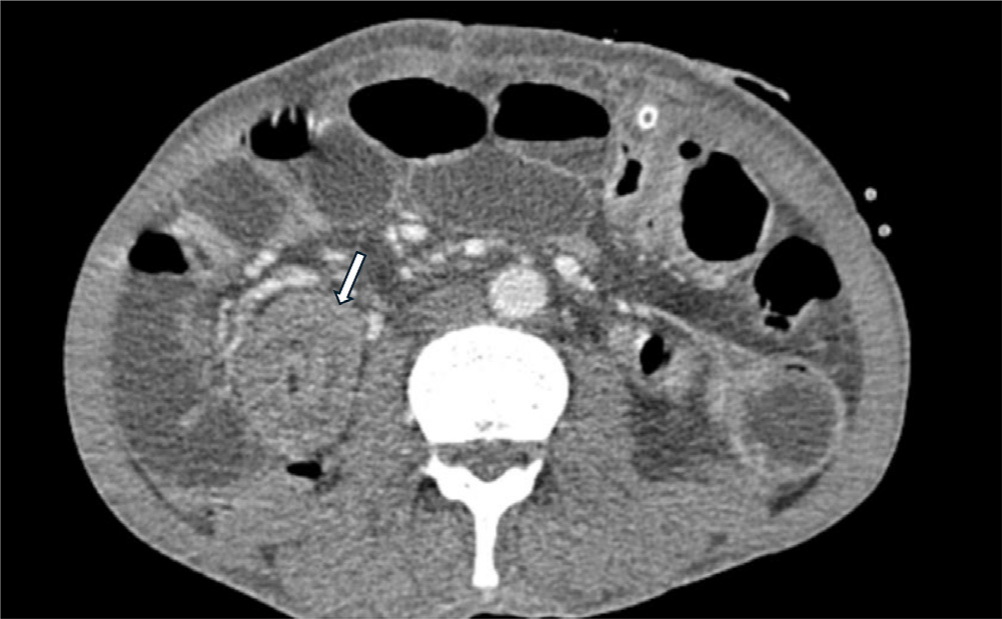
Axial abdominal CT scan showing the classic ``target sign'' (white arrow) consistent with intestinal intussusception around a feeding jejunostomy tube.

**Fig. 2:**
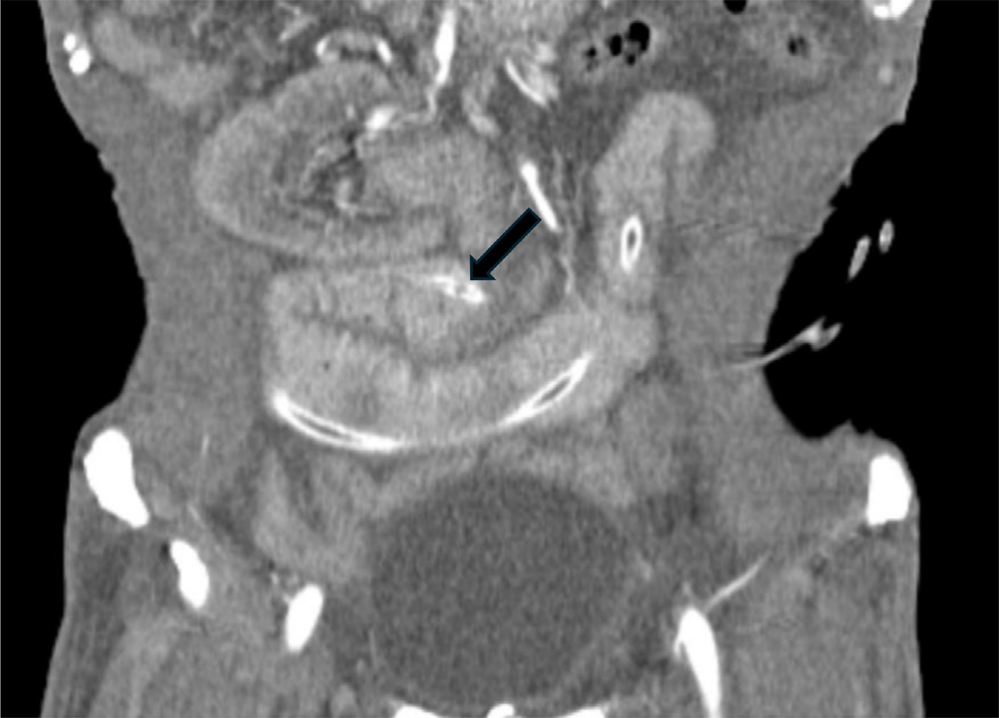
Coronal abdominal CT reconstructions showing intestinal intussusception around a feeding jejunostomy tube (black arrow).

Contrast-enhanced abdominal CT (axial slices, Fig. 1; coronal reconstructions, Fig. 2) showed marked small-bowel dilatation up to 36 mm in diameter with proximal air-fluid levels. A characteristic “target” or “bull’s-eye’’ sign was noted at the ileocecal valve, consistent with intussusception. The jejunostomy tube was clearly visualized within the intussuscepted segment, which measured approximately 10 cm in length and up to 49 mm in maximal thickness.

Surgical intervention was not pursued in this patient due to his cachectic condition, palliative status, and the high risk of recurrent obstruction. Instead, the attending physician-initiated corticosteroid therapy with close clinical monitoring. Under this conservative management, the patient experienced gradual improvement and regained normal bowel transit a few days after the CT examination.

**Fig. 3:**
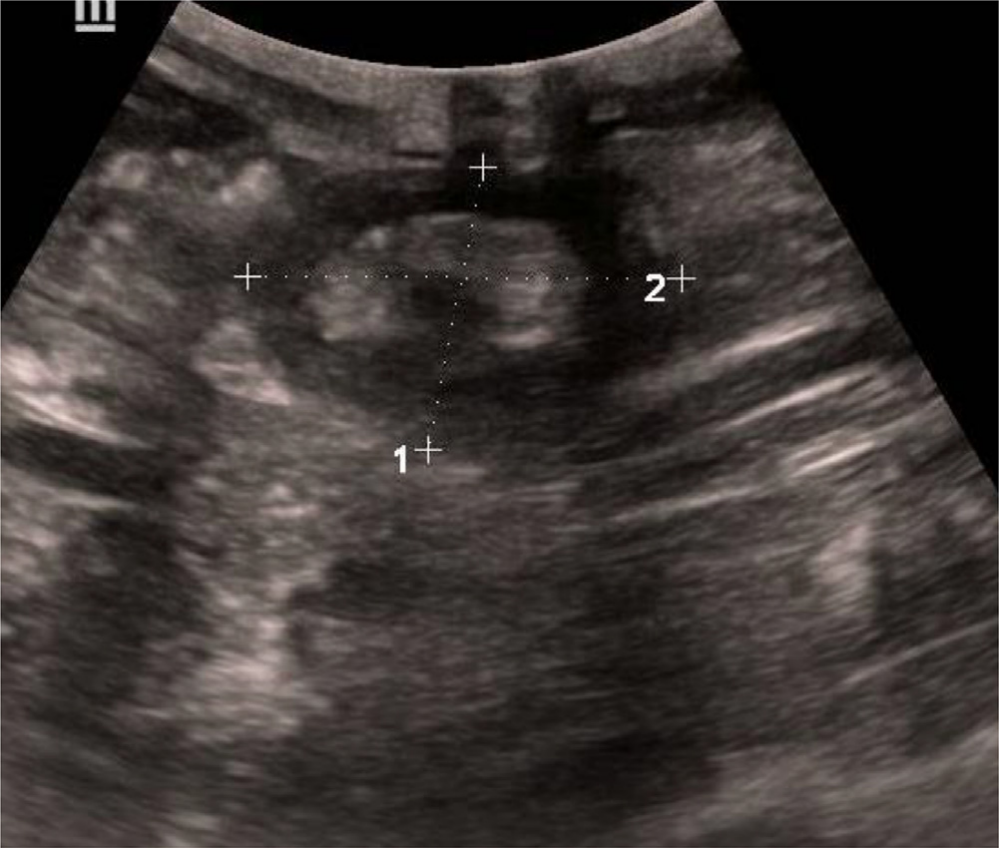
Ultrasound image showing the characteristic ``sandwich'' appearance of intestinal intussusception.

A follow-up CT scan showed resolution of the bowel obstruction, although the intussuscepted segment (intussusception mass) persisted. Subsequently, an abdominal ultrasound was performed (Fig. 3), revealing a hypoechoic, heterogeneous lesion in the right flank with a characteristic “target sign,” measuring 47 mm in diameter, consistent with acute intestinal intussusception.

## Discussion

Intestinal intussusceptions are classified according to their anatomical location within the gastrointestinal tract, including enteric, colocolic, ileocolic, colorectal, and rectorectal types [2]. They may occur in either an antegrade or retrograde manner. This condition typically presents around the fifth decade of life, with a balanced sex distribution (sex ratio 1:1) [2,3]. Jejunostomy-related intussusception is an exceptionally rare complication, accounting for less than 1% of all small bowel obstructions and approximately 5% of all intussusceptions. According to current literature, the most frequently reported complications associated with feeding jejunostomy include mechanical issues, bowel obstruction, and infectious processes [4].

In a study by Carucci et al. [5] involving 280 patients with feeding jejunostomies, complications occurred in 14% of cases (*n* = 40). These primarily included small bowel obstruction, intestinal strictures, and intra-abdominal fluid collections. Hematomas and small bowel intussusception were rare, occurring in only 1% of the cohort.

The pathophysiology of jejunostomy-related intussusception is not fully understood, but several mechanisms have been proposed. These include retrograde jejunal peristalsis, particularly in the context of recurrent vomiting, and the high infusion pressure during enteral feeding, which may cause the feeding tube to act as a stent, promoting bowel telescoping. Furthermore, patients requiring jejunostomy are often cachectic, with markedly reduced intra-abdominal fat (omentum and mesentery), allowing for increased mobility of bowel loops within the abdominal cavity. This anatomical predisposition may facilitate the development of intussusception [6].

Ultrasound can assist in the diagnosis by demonstrating the characteristic “sandwich sign” on longitudinal sections. However, abdominal computed tomography (CT) is considered the most sensitive imaging modality for confirming intestinal intussusception. CT can reveal high-grade small bowel obstruction proximal to a “target” or “bull’s-eye” appearance involving the jejunal loops [7]. The central hypodense area typically represents invaginated mesenteric fat containing mesenteric vessels, which are often enhanced after contrast administration. The presence of a hyperdense structure within the core of the intussuscepted segment is highly suggestive of an embedded jejunostomy tube.

From a therapeutic standpoint, reduction of intussusception prior to resection remains controversial. It should only be attempted when a benign lesion has been clearly identified preoperatively, or when resection would pose a significant risk of short bowel syndrome. This cautious approach is justified by the high incidence of associated malignancies in adult intussusception, which ranges from 1% to 40% [8].

## Conclusion

Jejunostomy tube-related intussusception is an exceptionally rare but potentially serious complication. Early recognition is crucial, as clinical symptoms are often nonspecific and can progress rapidly. Imaging, particularly CT scanning, plays a pivotal role in establishing the diagnosis. Surgical intervention remains the mainstay of treatment, especially in cases presenting with signs of bowel ischemia or obstruction.

## Ethics approval

Our institution does not require ethical approval for reporting individual cases.

## Patient consent

Written informed consent was obtained from the patient(s) for their anonymized information to be published in this article.
